# ﻿Parasitic crustaceans (Branchiura and Copepoda) parasitizing the gills of puffer fish species (Tetraodontidae) from the coast of Campeche, Gulf of Mexico

**DOI:** 10.3897/zookeys.1089.79999

**Published:** 2022-03-16

**Authors:** Ana Luisa May-Tec, Carlos Baños-Ojeda, Edgar F. Mendoza-Franco

**Affiliations:** 1 Instituto de Ecología, Pesquerías y Oceanografía del Golfo de México (EPOMEX), Avenida Héroe de Nacozari No. 480, CP. 24029, Universidad Autónoma de Campeche, San Francisco de Campeche, Campeche, Mexico Universidad Autónoma de Campeche Campeche Mexico

**Keywords:** Argulidae, aquaculture, biodiversity, Caligidae, Chondracanthidae, fisheries, interoceanic, Taeniacanthidae

## Abstract

New information on the marine parasitic crustaceans from the Campeche coast, Gulf of Mexico (GoM), can improve our baseline knowledge of the ecology of both the host and parasite by providing, for example, parameters of infection. Such knowledge is especially important for fish farming, so that appropriate quarantine measures can be established. Our aim was to morphologically identify the parasitic crustaceans infecting puffer fish of commercial importance in the coastal zone of Campeche, Mexico. We provide new information on four known species of parasitic crustaceans from 92 specimens representing five species of tetraodontid fish. The parasitic crustaceans *Argulus* sp. (Branchiura, Argulidae), *Caligushaemulonis* (Caligidae), *Pseudochondracanthusdiceraus* (Chondracanthidae), and *Taeniacanthuslagocephali* (Taeniacanthidae) (all Copepoda) were found on *Lagocephaluslaevigatus*, *Sphoeroidesnephelus*, *S.parvus*, *S.spengleri*, and *S.testudineus*. This study revealed the occurrence of *P.diceraus*, which is of importance in aquaculture, on *Sphoeroidesannulatus* in the Mexican Pacific. Additionally, our results and other documentary records provide the first evidence of the interoceanic occurrence of the same parasitic crustacean species in the south-southwest of Gulf of Mexico, the Atlantic Ocean, and the Pacific Ocean. Moreover, our study provides valuable information on the biodiversity of parasitic crustaceans present in the GoM on puffer fish which are of great commercial importance for human consumption, fisheries, and aquaculture.

## ﻿Introduction

Parasitic crustaceans are commonly known to cause serious lesions on farmed fish, causing destruction of gill tissue and favoring secondary infection, diseases, and massive mortality worldwide (Dezfuli et al. 2011; Aneesh et al. 2014; Misganaw and Getu 2016). Consequently, their presence represents a significant threat in aquaculture, with substantial potential economic losses. The probability of these organisms being introduced into farming systems is high, especially when an infected fish is caught from the wild and introduced into marine aquaculture (Bouwmeester et al. 2021).

In Mexico, studies on parasitic crustaceans belonging to Branchiura and Copepoda are scarce considering the high diversity of host species inhabiting the vast aquatic ecosystems (Morales-Serna et al. 2012). Knowledge of parasite diversity is an important step to understand how an ecosystem will respond to environmental stressors (Bennett et al. 2021). In particular, changes in the richness of parasitic species or individual parasites are indicative of environmental impact (Sures et al. 2017; Vidal-Martínez et al. 2019, 2022). The Gulf of Mexico (**GoM**) is characterized by activities such as overfishing and extraction of petroleum, which have a negative effect on biodiversity (Soto et al. 2014; Mendoza-Franco et al. 2018). However, this impact is difficult to estimate because of the limited biodiversity data.

The diversity of fish on the Campeche coast includes species such as puffer fish (Tetraodontiformes, Tetraodontidae) which are considered an economically important resource in southern Mexico and have the potential for aquaculture (Chávez-Sánchez et al. 2008). Notwithstanding this potential, knowledge of their parasitic crustaceans is rudimentary. This information is crucial to implement control tools and to create strategies for their safe management, especially for the commercial species.

Our aim was to identify morphologically the parasitic crustaceans infecting *Lagocephaluslaevigatus* (Linnaeus, 1766), *Sphoeroidesnephelus* (Goode & Bean, 1882), *S.parvus* (Shipp & Yerger, 1969), *S.spengleri* (Bloch, 1785), and *S.testudineus* (Linnaeus, 1758), all commercially important in the coastal zone of Campeche, Mexico. The geographic distribution of these copepods on puffer fish from the tropics is briefly discussed based on our findings and previous records.

## ﻿Material and methods

Using gill nets, we collected 92 puffer fish (69 *L.laevigatus*, 17 *S.spengleri*, 2 *S.testudineus*, 2 *S.parvus*, and 2 *S.nephelus*) from Seybaplaya, Campeche, southern Gulf of Mexico (19°42.580'N, 90°44.155'W), between November 2020 and April 2021. Fish were kept on ice for a maximum of 8 h and transported to the Laboratory of Aquatic Parasitology of EPOMEX (Instituto de Ecología, Pesquerías y Oceanografía del Golfo de México), Universidad Autónoma de Campeche (**UAC**). In the laboratory, we removed fish gills, placed them in bowls with 4% formaldehyde solution, and examined them under a Leica EZ4 stereomicroscope. We detached the parasitic crustaceans from gills by using fine needles, counted them, preliminarily identified them, fixed them in 70% alcohol, labeled them, and stored them in vials. We mounted individual specimens on slides and cleared them with glycerin at different concentrations (1:10, 1:5, 1:2). We examined dissected crustacean body parts following Humes and Gooding (1964). We identified crustaceans based on morphometrics using an Olympus microscope DM 2500. We follow the terminology of Ho (1970), Ho and Lin (2004), Lin and Ho (2006), and Møller et al. (2008) for *Caligus*, *Taeniacanthus*, *Pseudochondracanthus*, and *Argulus*, respectively. Measurements are provided in millimeters and expressed as a range. The prevalence, mean abundance, and intensity range are those proposed by Bush et al. (1997). We obtained synonyms for each host and crustacean species from FishBase (Froese and Pauly 2021) and World of Copepods (Walter and Boxshall 2021), respectively. Host body lengths are expressed as total length (**TL**). We deposited voucher specimens in the Colección Nacional de Invertebrados (**CNIN**), Universidad Nacional Autónoma de México, Mexico City, Mexico.

## ﻿Results

In total, 92 tetraodontid fish specimens were collected. The most abundant fish species was *L.laevigatus*, followed by *S.spengleri*, while *S.testudineus*, *S.parvus*, and *S.nephelus* were the least abundant species. Three parasitic crustacean species were found on *L.laevigatus* and a single species was found on the four *Sphoeroides* spp.

### ﻿Subclass Branchiura Thorell, 1864

**Order Arguloida Yamaguti, 1963**

**Family Argulidae Leach, 1819**

#### Genus *Argulus* Müller, 1785

##### ﻿ Argulus

Taxon classificationAnimaliaArguloidaArgulidae

sp.

B28DF940-A25B-5BEF-A749-DBEE5B4BEE82

###### Current host.

Smooth puffer *Lagocephaluslaevigatus* (Linnaeus) (Tetraodontidae) (TL: 27.5–47 cm).

###### Site of infection.

Gills.

###### Infection parameters.

Prevalence: 9% (six fish infected of 69 examined); mean abundance: 0.14 ± 1.03; intensity range: 1–3 individuals.

###### Source of current specimens.

Two voucher specimens deposited in the CNIN (171); collected on 30 November 2020.

###### Remarks.

**Figure 1.**
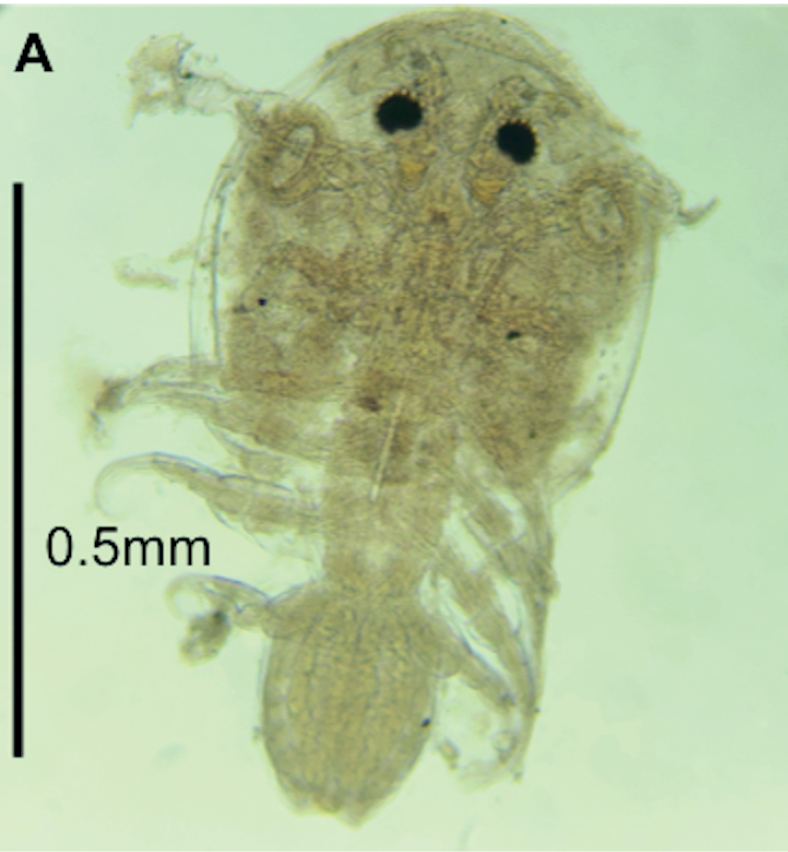
Parasitic crustacean *Argulus* sp. (Branchiura, Argulidae) on *Lagocephaluslaevigatus* from the Campeche coast, Gulf of Mexico.

These specimens are identified morphologically as *Argulus* sp., mainly by the shape and armature of cephalothoracic appendages, the presence of a modification of the first maxilla into a cup-like, stalked sucker, and legs (Møller et al. 2008). However, the specimens are larval stages, so their shape and size had not yet sufficiently developed for specific identification (Fig. 1). We report a species of *Argulus* from the coast of Campeche, Mexico, for the first time.

### ﻿Subclass Copepoda Milne Edwards, 1840

**Order Cyclopoida Burmeister, 1834**

**Family Chondracanthidae Milne Edwards, 1840**

#### Genus *Pseudochondracanthus* Wilson, 1908

##### ﻿ Pseudochondracanthus diceraus

Taxon classificationAnimaliaPoecilostomatoidaChondracanthidae

Wilson, 1908

C91E0491-6148-584E-88EB-7BB1D61A2982

###### Previous records.

*Sphoeroidesmaculatus* (Bloch & Schneider, 1801) (type host) from California (Wilson 1908); *S.nephelus* and *L.laevigatus* from Florida (Bere 1936); *S.spengleri* and *S.trichocephalus* (Cope, 1870) (as *S.tricocephalus*) from the coast of North Carolina to Florida, USA (Ho 1970); *S.annulatus* (Jenyns, 1842) (all Tetraodontidae) from the Pacific coast of Mexico (Morales-Serna et al. 2011).

###### Current hosts.

Southern puffer *Sphoeroidesnephelus* (TL: 21.6–21.6 cm), least puffer *S.parvus* (TL: 19.5–23 cm), bandtail puffer *S.spengleri* (TL: 13.9–24.0 cm), and checkered puffer *S.testudineus* (TL: 17.2–26.0 cm).

###### Site of infection.

Gills.

###### Infection parameters.

*Sphoeroidesnephelus*: prevalence: 100% (two fish infected of two examined); mean abundance: 5 ± 1.4; intensity range: 4–6 copepods. *S.parvus*: 100% (two fish infected of two examined); 2 ± 1.4; 1–3 copepods. *S.spengleri*: 89% (16 fish infected of 18 examined); 5.72 ± 4.89; 1–19 copepods. *S.testudineus*: 100% (two fish infected of two examined); 4 ± 1.4; 3–5 copepods.

###### Source of current specimens.

Ten voucher specimens (5 ♂, 5 ♀) from *S.spengleri* plus voucher and two specimens from *S.nephelus*, *S.parvus*, and *S.testudineus* deposited in the CNIN (172); collected on 27 April 2021.

###### Description

**Figure 2.**
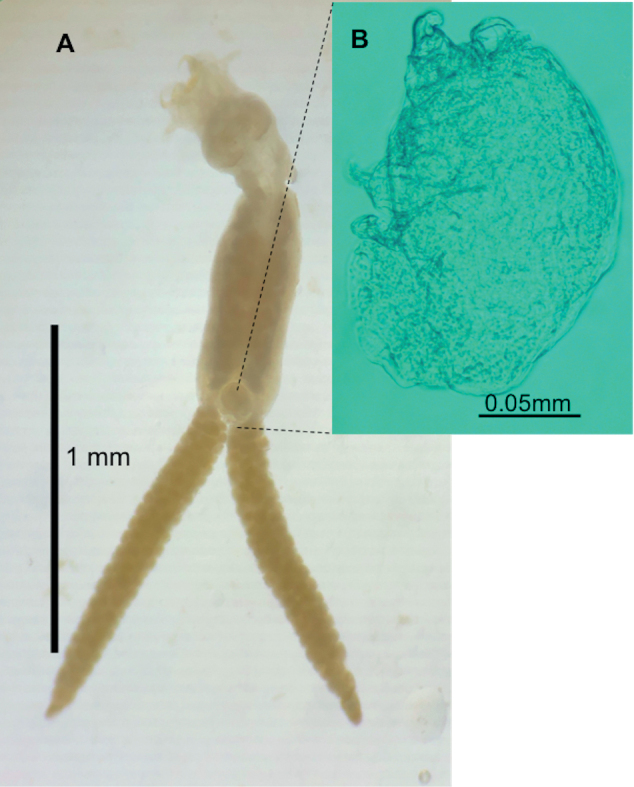
Parasitic copepods *Pseudochondracanthusdiceraus* (Copepoda, Chondracanthidae) on puffer fish from the Campeche coast, Gulf of Mexico **A** female **B** male.

**(based on 10 females and 7 males).** Adult female body 2.20–3.57 long. Head 0.75–0.87 long and 0.50–0.80 wide. Female genital complex elliptical, and entirely covered with small spines. Length of genital portion 1.34–2.35, and 0.56–1.0 wide. Length of egg strings 2.29–4.21 (Fig. 2A). Male body, 0.25–0.43 long and 0.12–0.20 wide (Fig. 2B). Urosome curved ventrally. Legs absent.

###### Remarks.

*Pseudochondracanthusdiceraus* was originally described by Wilson (1908) on the gills of common puffer *S.maculatus* from Massachusetts, USA. This parasitic copepod has also been reported in the same host from Massachusetts to North Carolina, in *S.spengleri* from North Carolina to Florida, in *S.trichocephalus* from the East coast of US, as well as in *L.laevigatus* and *S.nephelus* from the Gulf of Mexico, US coast (Wilson 1908; Bere 1936; Ho 1970). In Mexican Pacific waters, *P.diceraus* infected *S.annulatus* (Morales-Serna et al. 2011). *Pseudochondracanthusdiceraus* differs from the other congeneric species in having the trunk region covered with scale-like sclerotization (see Ho 1970: figs 236–251), which we clearly observed in the present specimens. Morphometrical comparison between the newly collected specimens and previous descriptions revealed insignificant differences. *Sphoeroidesparvus* and *S.testudineus* are new host records for *P.diceraus*, and Seybaplaya, Campeche, Mexico, is a new geographic record for this copepod species.

### ﻿Family Taeniacanthidae Wilson, 1911

#### Genus *Taeniacanthus* Sumpf, 1871

##### ﻿ Taeniacanthus lagocephali

Taxon classificationAnimaliaPoecilostomatoidaTaeniacanthidae

Pearse, 1952

2097A2F5-23C0-5CE6-8580-8B0C3D83A2EE

Irodes lagocephali Pillai, 1963: 124, fig. 7. Syn.
Taeniacanthus sabafugu Yamaguti & Yamasu, 1959: 102, pl. 4, figs 79, 89.

###### Previous records and localities.

*Lagocephaluslaevigatus* (type host) from Padre Island (Texas coast), Brazil, Alabama (Texas), Mississippi, and the Argentine Sea (Pearse 1952; Dojiri and Cressey 1987; Cantatore et al. 2012); *L.spadiceus* (Richardson, 1845) from Japan and the Mediterranean coast of Turkey (Yamaguti and Yamasu 1959; Özak et al. 2012); *L.lunaris* (Bloch & Schneider, 1801) from India (Pillai 1963); *L.inermis* (Temminck & Schlegel, 1850) from India (Umadevi and Shyamasundari 1980); *L.gloveri* (Abe & Tabeta, 1983) from Japan (Izawa 1986); *L.wheeleri* (Abe, Tabeta & Kitahama, 1984) from Taiwan (all Tetraodontidae) (Lin and Ho 2006).

###### Current host.

Smooth puffer *Lagocephaluslaevigatus* (Linnaeus) (Tetraodontidae) (TL: 20.3–48.5 cm).

###### Site of infection.

Gills.

###### Infection parameters.

Prevalence: 40% (28 fish infected of 69 examined); mean abundance: 1.10 ± 2.90; intensity range: 1–9 copepods.

###### Source of current specimens.

Ten voucher specimens (10 ♀) deposited in the CNIN (173); collected on 30 November 2020.

###### Description

**Figure 3.**
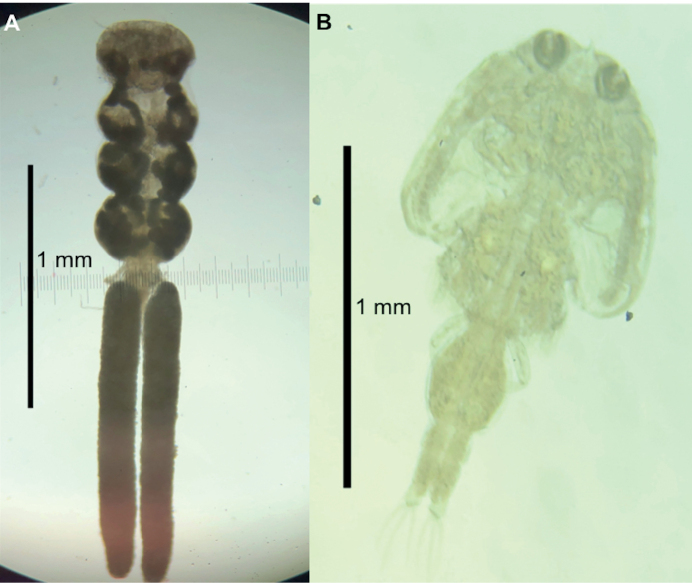
Parasitic copepods on *Lagocephaluslaevigatus* from the Campeche coast, Gulf of Mexico. **A***Taeniacanthuslagocephali***B***Caligushaemulonis*

**(based on 10 females)**. Total body length (not including setae of caudal rami) 2.52–3.33; cephalothorax length 0.54–0.76 and width 0.76–1.01 (Fig. 3A). Three thoracic segments as wide as cephalothorax (0.53 × 0.96; 0.53 × 0.90; 0.64 × 0.83). Urosome comprises five segments; genital complex (double-somite) much wider 0.26–0.35 than long 0.13–0.21. Anal somite with four interrupted rows of spinules and one row near the intersection of each caudal ramus. Caudal ramus (0.050 × 0.04) bearing six setae: two long apical, one short subterminal at inner and outer corners, one short dorsal, and one short seta on outer margin near center. Maxillary hook large, slender, slightly curved, located on the anteroventral surface of cephalothorax to junction of first and second segments of first antenna. First maxillae with two pinnate setae. Second maxillae bi-segmented, bearing two terminal spiniform processes on second segment. Maxilliped three-segmented; basal segment unarmed; second segment armed with two basal setae; and terminal segment forming a claw curved with serrations on convex margin of distal portion.

###### Remarks.

Pearse (1952) originally described *T.lagocephali* infecting the gills of *L.laevigatus* from Padre Island, Texas. Yamaguti and Yamasu (1959) reported it as *Taeniacanthussabafugu* from *L.spadiceus* from Japan, and Pillai (1963) described it as *Irodeslagocephali* from *L.lunaris* and *L.inermis* from India. Subsequently, Ho (1970) recognized all these copepod species from *L.spadiceus*, *L.lunaris*, and *L.inermis* as synonyms of *T.lagocephali*. This parasitic copepod is characterized by having a cephalothorax with three thoracic segments equal in width, a maxilliped with a terminal curving claw, and a digitiform process (Dojiri and Cressey 1987: fig. 33). We also observed these morphological characteristics in our specimens, and they are consistent with the original description and the specimens redescribed by Dojiri and Cressey (1987), Lin and Ho (2006), and Özak et al. (2012). However, some metric differences were observed in the total length between the newly collected specimens from *T.lagocephali* and those reported from *L.spadiceus* by Özak et al. (2012) from the Mediterranean coast of Turkey (2.95 mm vs 1.9 mm). These are probably due to intraspecific variation over large geographic distances or from effects of hosts; that is, host body size is one of five alternative hypotheses which can potentially generate a geographic pattern in parasite body size, while following the Bergmann’s rule suggested by Poulin (2021). Studies have demonstrated a positive relationship between the parasite body size and the host body size (Poulin et al. 2003). So, *L.laevigatus* reaches sizes larger than *L.spadiceus* (100 cm vs 37.4 cm), and this can explain the metric differences of *T.lagocephali* found on these hosts. Furthermore, Lin and Ho (2006) reported four setae on the third segment of the antennule, while Dojiri and Cressey (1987) and Özak et al. (2012) reported five on the same segment, the number we observed in our specimens. Additionally, the number of rows of spinules on the ventral surface (three) of the anal segment reported by Lin and Ho (2006) contrast with the four rows of spinules reported by Dojiri and Cressey (1987), Özak et al. (2012), and in our material. Another explanation for these morphological differences could be result of a phenotypic variation in this species, and a phylogenetic study comparing these morphologic differences may contribute to a better understanding of this variation.

*Taeniacanthuslagocephali* has been reported on *Lagocephalus* spp. from the Oriental region (Japan and Taiwan), the Ethiopian region (West Africa), the Nearctic region (GoM coast of Mississippi, Alabama, and Texas), and the Neotropical region (Brazil) (Pearse 1952; Yamaguti and Yamasu 1959; Pillai 1963; Umadevi and Shyamasundari 1980; Izawa 1986; Dojiri and Cressey 1987; Lin and Ho 2006; Cantatore et al. 2012; Özak et al. 2012). The wide distribution of this parasite could be attributed to its host specificity to the genus *Lagocephalus* and its capacity to exploit this host genus in different geographic ranges. Host specificity is a determinant key in how the parasites can be established into new areas (Poulin et al. 2011). In Mexico, Taeniacanthidae has been represented only by *Taeniacanthodesdojirii* Braswell, Benz & Deets, 2002 in the ray *Narcineentemedor* (Narcinidae) from Bahía de Los Angeles, Santa Rosalía, Gulf of California (Braswell et al. 2002). Our present record is the first occurrence of *T.lagocephali* on *L.laevigatus* from the GoM. Together with the only species previously reported (Braswell et al. 2002), the number of species of Taeniacanthidae in Mexico is now two.

### ﻿Order Siphonostomatoida Burmeister, 1835

**Family Caligidae Burmeister, 1835**

#### Genus *Caligus* Müller, 1785

##### ﻿ Caligus haemulonis

Taxon classificationAnimaliaSiphonostomatoidaCaligidae

Krøyer, 1863

F1C8AE96-5C50-5474-9A57-05CB2AE1F986

###### Previous records.

See Table 1.

###### Current host.

Smooth puffer *Lagocephaluslaevigatus* (Linnaeus) (Tetraodontidae) (TL: 20.3–48.5 cm).

###### Site of infection.

Gills.

###### Infection parameters.

Prevalence: 49% (34 fish infected of 69 examined); mean abundance: 3.63 ± 7.45; intensity range: 1–30 copepods.

###### Source of current specimens.

Ten voucher specimens (5 ♀, 5 ♂) deposited in the CNIN (174); collected on19 January 2021.

###### Description

**(based on 10 females and 10 males).** Adult female body caligiform, 2.70–3.30 long. Cephalothorax 1.50–1.80 long and 1.43–1.63 wide. Female genital complex longer than wide, lacking distinct posterolateral lobes (Fig. 3B). Caudal rami armed with five pinnate setae. Female antenna with distal claw strongly curved. Sternal furca of female with incurved tines. Maxilliped with smooth myxal margin, with a tiny process on inner margin of the claw. Male 2.10–2.50 long. Cephalothorax 1.10–1.30 long and 0.95–1.47 wide. Sternal furca more incurved in males. Male maxilliped with large, acutely pointed process on myxal margin opposing tip of claw. In both sexes, post-antennal process large and strongly curved. Last exopodal segment of leg I with one long seta at inner distal angle, three distal spines, and posterior margin a single naked vestigial seta. Outer margin of second endopodal segment of leg II with setules. Leg IV with robust first exopodal segment bearing marginal setule; second segment with well-developed spines.

###### Remarks.

Currently, the genus *Caligus* comprises more than 270 valid species worldwide (Walter and Boxshall 2021) on a wide variety of marine fish. In Mexican waters 31 species of *Caligus* are known, 21 from the Pacific, seven from GoM, and three from both the Atlantic and Pacific coasts (Morales-Serna et al. 2014). *Caligushaemulonis* has been recorded on the Atlantic coast from Florida to Brazil on a wide variety of fish families and only one species of ray (*Aetobatusnarinari*) on the Campeche coast (Rodríguez-Santiago et al. 2016) (Table 1). The morphologic characteristic of our specimens coincide with the original description of *C.haemulonis* (Krøyer 1863; Boxshall and El-Rashidy 2009).

**Table 1.** Previous records of *Caligushaemulonis* on a wide variety of fish teleost (14 families) and one elasmobranch species having cosmopolitan distribution.

Host	Locality	Reference
**Ariidae**		
*Ariopsisfelis* (Linnaeus, 1766) (as *Hexanematichthysfelis*, *Galeichthysfelis* and *Ariusfelis*)	Atlantic coast of USA	Wilson 1908
*Aspistorluniscutis* (Valenciennes, 1840) (as *Ariusluniscutis*, *Notariusluniscutis*)	Brazil	Luque and Tavares 2007
*Bagremarinus* (Mitchill, 1815) (as *Felichthysmarinus* and *Bagremarina*)	Atlantic coast of USA	Wilson 1908
*Carlariusheudelotii* (Valenciennes, 1840) (as *Ariusheudelotii*)	Africa, Mediterranean	Brian 1924
*Genidensbarbus* (Lacepède, 1803)	Brazil	Luque and Tavares 2007
**Carangidae**		
*Campogrammaglaycos* (Lacepède, 1801) (as *Lichiavadigo*)	Mediterranean	Brian 1924
*Caranxcrysos* (Mitchill, 1815)	Louisiana	Causey 1953
*Caranxrhonchus* Geoffroy Saint-Hilaire, 1817 (as *Caranxangolensis*)	Africa South	Capart 1959
*Trachurustrachurus* (Linnaeus, 1758)	Africa South	Capart 1959
**Engraulidae**		
*Anchoamarinii* Hildebrand, 1943	Brazil	Luque and Tavares 2007
**Ephippidae**		
*Chaetodipterusfaber* (Broussonet, 1782)	Brazil, Florida	Cezar and Luque 1999
**Haemulidae**		
*Anisotremusvirginicus* (Linnaeus, 1758)	Belize	Cressey 1991
*Haemuloncarbonarium* Poey, 1860	Belize	Cressey 1991
*Haemulonmacrostomum* Günther, 1859	Belize	Cressey 1991
*Haemulonplumierii* (Lacepède, 1801)	Belize	Cressey 1991
*Haemulonsciurus* (Shaw, 1803) (type host)	Danish West Indies of Insular Caribbean	Krøyer 1863
*Haemulonsteindachneri* (Jordan & Gilbert, 1882)	Brazil	Luque and Takemoto 1996
*Orthopristisruber* (Cuvier, 1830)	Brazil, Florida	Luque and Takemoto 1996
*Plectorhinchusmediterraneus* (Guichenot, 1850) (as *Diagrammamediterraneum*)	Africa, Mediterranean	Brian 1924
**Kyphosidae**		
*Girellatricuspidata* (Quoy & Gaimard, 1824)	Australia	Boxshall and El-Rashidy 2009
**Monacanthidae**		
*Aluterusschoepfii* (Walbaum, 1792) (as *Aleuterusschoepfi*)	Florida	Cressey 1991
**Myliobatidae**		
*Aetobatusnarinari* (Euphrasen, 1790) (as *Stoasodonnarinari*)	Tabasco to Campeche coast Gulf of Mexico	Rodríguez-Santiago et al. 2016
**Polynemidae**		
*Polydactylusquadrifilis* (Cuvier, 1829)	Africa	Oldewage and Avenant-Oldewage 1993
**Pomatomidae**		
*Pomatomussaltatrix* (Linnaeus, 1766) (as *Temnodonsaltator*)	Mediterranean	Brian 1924
**Rachycentridae**		
*Rachycentroncanadum* (Linnaeus, 1766)	USA	Williams and Bunkley-Williams 1996
**Sciaenidae**		
*Argyrosomusregius* (Asso, 1801) (as *Sciaenaaquila*)	Mediterranean	Brian 1924
*Bairdiellachrysoura* (Lacepède, 1802)	Florida	Cressey 1991
*Menticirrhusamericanus* (Linnaeus, 1758) (as *Menthicirrhusamericanus*)	Brazil, Florida	Chaves and Luque 1999
*Micropogoniasfurnieri* (Desmarest, 1823) (as *Micropogonfurnieri*)	Brazil	Alves and Luque 2000
*Paralonchurusbrasiliensis* (Steindachner, 1875)	Brazil	Ribeiro et al. 2002, Luque et al. 2003
*Pogoniascromis* (Linnaeus, 1766)	Florida	Bere 1936
*Pseudotolithusmoorii* (Günther, 1865) (as *Corvinacamaronensis*)	Africa South	Capart 1959
*Sciaenaumbra* Linnaeus, 1758 (as *Corvinanigra*)	Mediterranean	Brian 1924
*Sciaenopsocellatus* (Linnaeus, 1766) (as *Sciaenopsocellata*)	Louisiana	Causey 1953
*Umbrina* sp.	Africa South	Capart 1959
**Serranidae**		
*Centropristisstriata* (Linnaeus, 1758)	Florida	Wilson 1908
**Sparidae**		
*Archosargusprobatocephalus* (Walbaum, 1792)	Florida	Cressey 1991
*Archosargusrhomboidalis* (Linnaeus, 1758)	Brazil	Cordeiro and Luque 2005
*Dentex* sp.	Africa, Mediterranean	Brian 1924
*Dentexgibbosus* (Rafinesque, 1810) (as *D.filosus*)	Africa South	Capart 1959
*Pagrus* sp.	Africa, Mediterranean	Brian 1924
*Pagruspagrus* (Linnaeus, 1758)	Brazil	Paraguassú et al. 2002
**Triglidae**		
*Prionotuspunctatus* (Bloch, 1793)	Brazil	Bicudo et al. 2005
*Triglalyra* Linnaeus, 1758	Africa South	Capart 1959

*Caligushaemulonis* and 13 other parasitic copepods are included in the *Caligusproductus* group; they are characterized by loss of two and reduction or loss of the third of the three plumose setae on the distal exopod segment of the first swimming leg (see Boxshall and El-Rashidy 2009: figs 5, 6). In particular, *C.haemulonis* lacks the plumose setae and has a tiny naked vestigial seta on the posterior margin of the distal exopodal segment of leg I, as seen in the present specimens and the description of Cressey (1991), who was the first to observe this character. We found differences in the body length between our specimens and those reported by Suárez-Morales et al. (2010): females 2.70–3.30 mm vs 3.1–3.2 mm from *H.sciurus* and *H.plumierii* (Haemulidae) in Suárez-Morales et al. (2010) from Mexico, 3.56 mm in Cressey (1991), 3.33–3.92 mm on *Orthopristisruber* and *Haemulonsteindachneri* (all Haemulidae) from Brazil in Luque and Takemoto (1996), and 2.96–3.92 mm in Boxshall and El-Rashidy (2009) from Brazil; males measured 2.10–2.50 mm vs 1.75–1.81 mm from haemulids in Suárez-Morales et al. (2010) from Mexico; 1.86–3.26 mm in Cressey (1991) from Florida and Boxshall and El-Rashidy (2009) from Brazil. The variability in the size of parasites can be attributed to their stage of maturity, because the measurements of the collected specimens (females and males) are within the size range reported in previous studies (Cressey 1991; Luque and Takemoto 1996; Boxshall and El-Rashidy 2009; Suárez-Morales et al. 2010). The characteristics of the female sternal furca (i.e., tines slightly thinner) in our specimens and those reported by Suárez-Morales et al. (2010) are identical (see Suárez-Morales et al. 2010: 169, 171, figs 1, 2). *Caligushaemulonis* is an ectoparasite on a wide variety of teleosts (Margolis et al. 1975; Cressey 1991; Chaves and Luque 1999; Boxshall and El- Rashidy 2009; Suárez-Morales et al. 2010) and some elasmobranchs (Kabata 1979; Tang and Newbound 2004; Rodríguez-Santiago et al. 2016). Our material represents a new host record of this parasitic copepod species in the Mexican GoM.

## ﻿Discussion

This study represents the first records of branchiuran and copepod parasites on tetraodontids of the Campeche coast. Previous records from this area mentioned the presence of 15 species of copepods parasitizing elasmobranchs; some have also been reported for other elasmobranch species worldwide (Rodríguez-Santiago et al. 2016). However, of these species only one copepod (*C.haemulonis*) coincides with those reported in our study. All records we have reported here are new host records or new geographic records. Below, we briefly discuss the distribution of the puffer fish hosts that these crustaceans parasitize.

Members of *Argulus* have a wide range of fish hosts and environments (freshwater and marine) around the world. In the GoM, 10 species have been reported, especially from the north-northwest coast of the USA (Poly 2009). In Mexican waters, six species of *Argulus* are recorded: *Arguluschromidis* Krøyer, 1863 and *Argulusrhamdiae* Wilson, 1936 on *Rhamdiaguatemalensis* Günther, 1864 from Yucatán (Wilson 1936), *Argulusflavescens* Wilson, 1916 on *Ariopsisassimilis* Günther, 1864 (as *Ariusassimilis*) from Chetumal (Suárez-Morales et al. 1998), *Argulusmexicanus* Pineda, Paramo & del Rio, 1995 on *Atractosteustropicus* Gill, 1863 from Tabasco (Pineda et al. 1995), *Argulusambystoma* Poly, 2003 on *Ambystomadumerilii* Dúges, 1870 from Lake Patzcuaro, Michoacan (Poly 2003), and *Argulusyucatanus* Poly, 2005 on *Mayaherosurophthalmus* Günther, 1862 (as *Cichlasomaurophthalmus*) from Yucatán (Poly 2005). All these records are from freshwater fishes, except for *A.flavescens*, which occurs in freshwater, marine, and brackish-water fishes (Suárez-Morales et al. 1998). These infections have rarely been found to have severe effects on natural fish populations (Taylor et al. 2005). However, their presence is important, especially in fishes with aquaculture potential, such as puffer fish. These ectoparasites cause dermal damages that promotes secondary infections and, in severe cases, high mortality in aquaculture systems where these types of infections are intensified (Patra et al. 2016). Additional adult specimens of *Argulus* sp. are necessary to determine the species.

The morphological characteristics of specimens *Taenicanthuslagocephali* in *L.laevigatus* collected here agree with the original description and redescription of specimens from North and South America (Pearse 1952; Dojiri and Cressey 1987). The geographic proximity of GoM to the Atlantic Ocean and the wide host distribution could explain the morphological similarity of our specimens to Atlantic populations. However, some differences have been found with the description of *T.lagocephali* from the Mediterranean coast and Taiwan. These could probably be attributed to intraspecific variation in the geographic distance of the hosts. We believe that future studies incorporating phylogenetic analyses are necessary to confirm the identity and to accurately assess the distribution of these species, as well as to understand their host specificity.

Cressey (1991) and Suárez-Morales et al. (2010) have suggested that in the Mexican Caribbean, despite its high ichthyological diversity, haemulids are the preferred hosts of *C.haemulonis*, with a prevalence ranging from 6 to 13%. However, we found a higher prevalence in *L.laevigatus* (> 40%), which suggests that *C.haemulonis* does not present a host preference, as proposed. However, to affirm this assumption, a study is necessary that includes the haemulids as the abundant fishes on the Campeche coast (Crespo-Guerrero et al. 2019; Borges-Ramírez et al. 2020). *Caligushaemulonis* has a broad host range; this characteristic is especially important to fish farming because the introduction of infected wild fish could cause its transmission to new hosts. Therefore, the record of *C.haemulonis* in puffer fishes from southern Mexico accounts for the geographic range of this parasitic copepod and its expansion to new hosts in the region. In addition, this information could contribute to implementation of measures to prevent its transmission—that is, quarantine of wild fish—to farmed fish such as puffer fish.

With exception of *L.laevigatus*, all other species of *Sphoeroides* examined were parasitized with *P.diceraus*. This suggests that *Sphoeroides* spp. could be the preferred hosts of this parasite. Future examination of other hosts in the same area is necessary to confirm this assumption. This is the first record of *P.diceraus* parasitizing a puffer fish from the GoM. In previous studies on parasitofauna of puffer fishes from the southern of GoM (Vidal-Martínez and Mendoza-Franco 2008; Pech et al. 2009), this copepod was not reported. Special attention should be paid to the presence of *P.diceraus*, which has caused high mortality in the culture of *S.annulatus* (Fajer-Ávila et al. 2011).

Our findings suggest that the composition of ectoparasites on puffer fishes from the Campeche coast differs from that reported for the Yucatán Peninsula by Vidal-Martínez and Mendoza-Franco (2008) and Pech et al. (2009), despite the wide distribution of host species. These differences in ectoparasite composition might be due to the physicochemical (water quality, nutrients, and water flow rates) and biological characteristics of the regions along the south-southwest coast from Tabasco to Campeche, and along the south-southeast coast in the Yucatán Peninsula. This hypothesis has been partially tested through a comparative study of the parasitofauna of flounder fish from the Yucatán Peninsula (i.e., *Syaciumpapillosum* and *Syaciumgunteri*) (Vidal-Martínez et al. 2019). Vidal-Martínez et al. (2019) found variation in the parasite composition associated with environmental variables, suggesting the existence of two subregions in the Yucatán Peninsula (the Campeche Sound and the Yucatán Shelf). However, a comparative study of the parasitofauna of *Sphoeroides* spp. between the two regions, considering the ecological data, could contribute to a better understanding of the differences.

The occurrence of *P.diceraus* in the Pacific and along the Campeche coast is noteworthy. *Pseudochondracanthusdiceraus* was originally described in commercially important fish *Sphoeroidesmaculatus* from the Atlantic and Pacific coast of the USA (Wilson, 1908); however, *S.maculatus* is a fish native to the North Atlantic. Its presence in the Pacific is remarkable and it is tempting to speculate that its presence there is the result of translocation of parasites associated with the natural distribution of their hosts or a consequence of anthropogenic activities (i.e., host introductions; Goedknegt et al. 2016; Paredes-Trujillo et al. 2020). However, the distribution mechanisms of copepod species are not well understood, and information has mainly focused on taxonomy. Nevertheless, *P.diceraus* has previously been reported on *S.spengleri* and *S.nephelus* from Florida and the US Gulf Coast (Wilson 1908; Bere 1936; Ho 1970). The GoM is part of the geographical range of this puffer fish, so the presence of *P.diceraus* on the Campeche coast can be attributed to the natural distribution of these *Sphoeroides* spp.

On the other hand, the broad geographic distribution of *P.diceraus* could be explained by a hypothesis suggested by Kritsky (2012) who suggested that the geological formation of the Panamanian isthmus approximately 3.2 Ma ago divided ancestral hosts as well as their monogeneans into eastern Pacific and western Atlantic populations.

Therefore, the geographical distribution of both parasitic crustacean and the monogeneans could be attributed to the dispersal capabilities of their hosts (Skern-Mauritzen et al 2014; Paladini et al 2021). Therefore, we suggest that parasitic crustaceans could have undergone a similar distribution and speciation. However, a phylogenetic hypothesis based on molecular and morphological data for these parasitic crustaceans on puffer fish would provide the needed information on their diversification as evidence of a speciation process associated with geological history or the influence of ecological factors; this would provide a more comprehensive understanding of the biogeographical distribution of parasitic crustaceans in the tropics.

## ﻿Conclusions

We have revealed the occurrence of marine parasitic crustaceans of importance for fish aquaculture on the Campeche coast. We have deduced that the composition of ectoparasites on puffer fishes of the Campeche coast and Yucatán Peninsula differ and this difference is associated with differing environmental characteristics of each area, despite the geographical proximity. Our results represent only a small fraction of diversity of parasitic crustaceans present in the GoM, but they provide valuable new information on the geographical distribution and hosts in the region (i.e., the occurrence of an interoceanic copepod species), which is especially relevant aquaculture. To explore host specificity, the ecological and parasite-host interaction associated with their distribution, studies focusing on morphology and phylogenetics are essential.

## Supplementary Material

XML Treatment for Argulus

XML Treatment for Pseudochondracanthus diceraus

XML Treatment for Taeniacanthus lagocephali

XML Treatment for Caligus haemulonis
